# Antitumor Effects of Esculetin, a Natural Coumarin Derivative, against Canine Mammary Gland Tumor Cells by Inducing Cell Cycle Arrest and Apoptosis

**DOI:** 10.3390/vetsci10020084

**Published:** 2023-01-22

**Authors:** Jawun Choi, Min-Jae Yoo, Sang-Youel Park, Jae-Won Seol

**Affiliations:** College of Veterinary Medicine, Jeonbuk National University, Iksan 54596, Jeollabuk-do, Republic of Korea

**Keywords:** canine mammary gland tumors, esculetin, apoptosis, cell cycle arrest

## Abstract

**Simple Summary:**

Canine mammary gland tumors (CMTs) are the most common type of tumor in female dogs, and approximately 50% of them are diagnosed as malignant. Currently, several chemotherapeutic agents have been applied to treat those CMTs that are difficult to remove by surgery, due to metastasis. However, it is necessary to discover more safe and specific drugs for the treatment of CMTs. This research investigates the anticancer effects of esculetin, a natural coumarin derivative, and its underlying mechanisms on CMT cell lines, CMT-U27 and CF41.mg. Esculetin remarkably inhibited the viability and migration of both cell lines. Esculetin treatment activated the protein expression of caspase 3, a typical marker of apoptosis, leading to apoptotic cell death. Furthermore, esculetin promoted cell cycle arrest in both cell lines. Interestingly, esculetin has potently reduced the protein expression of CDK4 and cyclin D1, regulators of G1/S transition, in both cell lines; however, cell cycle arrest was caused at a different phase in both cell lines, i.e., at the G0/G1 phase in CMT-U27 cells and the S phase in CF41.mg cells. These results demonstrate the anticancer effect of esculetin and lay a theoretical foundation for in vivo experiments and clinical trials.

**Abstract:**

Mammary gland tumors are the most common neoplasms in female dogs, of which 50% are malignant. Esculetin, a coumarin derivative, reportedly induces death in different types of cancer cells. In this study, we explore the anticancer effects of esculetin against CMT-U27 and CF41.mg canine mammary gland tumor cells. Esculetin significantly inhibited the viability and migration of both CMT-U27 and CF41.mg cells in a dose- and time-dependent manner. Flow cytometric analysis and terminal deoxynucleotidyl transferase dUTP nick-end labeling assay revealed increased numbers of annexin-V-positive cells and DNA fragmentation. Furthermore, a cell cycle analysis demonstrated that esculetin blocked the cell progression at the G0/G1 phase and the S phase in CMT-U27 and CF41.mg cells. These results were supported by a Western blot analysis, which revealed upregulated protein expression of cleaved caspase-3, a marker of apoptosis, and downregulated cyclin-dependent kinase 4 and cyclin D1 protein, the cell cycle regulators. In conclusion, this novel study proves that esculetin exerts in vitro antitumor effects by inducing apoptosis and cell cycle arrest in canine mammary gland tumors.

## 1. Introduction

Canine mammary gland tumors (CMTs) are one of the most frequent tumors in intact female dogs, and approximately 50% of these tumors are diagnosed as malignant [1,2]. Currently, surgical resection is the primary therapeutic option for CMTs; however, recurrence and/or metastasis remain challenging in patients with CMTs [3,4]. Therefore, to improve the poor prognosis after surgery, adjuvant therapy, including immunotherapy, radiotherapy, and chemotherapy has been often used. A recent study demonstrated the antitumor efficacy and safety of intratumoral empty cowpea mosaic virus-immunotherapy in canine inflammatory mammary cancer (IMC) patients [5]. Another study reported that radiotherapy in combination with anti-angiogenic drugs showed a longer time to progression and survival time in dogs with IMC [6]. In chemotherapy, one of the major strategies is that anticancer drugs for humans are applied to treat dogs with CMTs. Rivoceranib, a novel tyrosine kinase inhibitor, exhibited antiproliferative effects and induced cell cycle arrest in CMT cell lines. Rivoceranib also decreased the average tumor volume in in vivo xenograft models [7]. Meloxicam, a non-steroidal anti-inflammatory drug, significantly suppressed cell migration and invasion by downregulating β-catenin expression in CF41.mg canine mammary-carcinoma cells [8]. In addition, lipid-lowering drugs, namely atorvastatin and fluvastatin, exerted anticancer effects in CMT9 and CMT47 cell lines by inducing apoptosis and cell cycle arrest [9]. However, the same as in humans, chemotherapy can cause severe pain and side effects in canines. Hence, this necessitates the discovery of safe and effective drugs for the treatment of CMTs.

Natural derivatives have therapeutic and pharmacological properties, including antioxidant, anti-inflammatory, and anticancer activity [10,11,12]. Compared with synthetic compounds, natural compounds may be ideal choices for cancer treatment because of their unique biological properties and reduced side effects [13]. In veterinary oncology, researchers have reported on the anticancer effects of natural derivatives, such as the latex of *S. grantii* on metastatic canine prostate-cancer cells [14], berberine, and *Euphorbia royleana* on canine mammary-gland cancer cells [15,16]. Esculetin, also termed as 6,7-dihydroxycoumarin, is a natural coumarin derivative present in various plants, such as *Aesculus hippocastanum*, *Fraxinus rhynchophylla*, and *Artemisia capillaris* [17,18]. It reportedly possesses pleiotropic biological and pharmacological activities, including antiedema, anti-inflammatory, antioxidant, and antitumor effects [19,20,21,22]. Several studies have demonstrated the inhibitory effects of esculetin against human cancers, such as cervical cancer [23], oral cancer [24,25], liver cancer [26], colon cancer [27], and renal cancer [28].

However, research has not yet been conducted to identify the effects of esculetin on CMT cells and its underlying mechanisms. Therefore, this study examines the inhibitory effect of esculetin on cell viability and migration in CMT cells and explores its effects on apoptosis and cell cycle arrest.

## 2. Materials and Methods

### 2.1. Cell culture and Reagents

CMT-U27 and CF41.mg cell lines were purchased from ATCC (Manassas, VA, USA). The cells were cultured in Roswell Park Memorial Institute 1640 medium (CMT-U27 cells) or Dulbecco’s modified Eagle’s medium (CF41.mg cells; Gibco, Grand Island, NY, USA) and supplemented with 10% fetal bovine serum (Atlas Biologicals, Fort Collins, CO, USA), 100 U/mL of penicillin, and 100 μg of streptomycin (Sigma-Aldrich, St. Louis, MO, USA). Canine aortic endothelial cells (CnAOECs) and their growth medium were purchased from Cell Applications, Inc. (San Diego, CA, USA). All cells were grown at 37 °C with 5% CO_2_. Esculetin (Sigma-Aldrich) was dissolved in dimethyl sulfoxide to make 500 mM stock solution, which was subsequently diluted with the media.

### 2.2. Crystal Violet Staining

CMT-U27 and CF41.mg cells were seeded in 12-well plates overnight and treated with different concentrations of esculetin (0 mM, 0.25 mM, 0.5 mM, and 1 mM) for 24 h and 48 h. After treatment, the viable cells were stained with crystal violet, rinsed in tap water, and dried. These cells then were lysed in 1% sodium dodecyl sulfate solution. We recorded the absorbance at 550 nm using a microplate reader (Spectramax M2; Molecular Devices, CA, USA).

### 2.3. 3-(4,5-dimethylthiazol-2-yl)-2,5-diphenyltetrazolium bromide (MTT) Assay

We determined cell viability by a 3-(4,5-dimethylthiazol-2-yl)-2,5-diphenyltetrazolium bromide (MTT) assay. CMT-U27 and CF41.mg cells were cultured overnight in 24-well plates and treated with different concentrations of esculetin (0 mM, 0.25 mM, 0.5 mM, and 1 mM). Following 24 h and 48 h, the media was carefully removed and 300 μL of MTT solution (0.5 mg MTT/mL medium) was added to each well; the plates were incubated for 2 h at 37 °C. Consequently, the medium was replaced with 500 μL dimethyl sulfoxide, and the plates were shaken for 10 min. Subsequently, 200 μL of the mixed extraction solution was transferred to a 96-well microplate. We measured the absorbance at 570 nm using a microplate reader (Molecular Devices).

### 2.4. Lactate Dehydrogenase (LDH) Release Assay

We measured the lactate dehydrogenase (LDH) levels released from the damaged cells into the culture medium using a cytotoxicity detection kit (Takara Bio Inc., Shiga, Japan) according to the manufacturer’s instructions. Briefly, the cells were cultured in 24-well plates overnight and treated with or without esculetin for 24 h and 48 h. Following treatment, the culture medium was collected and centrifuged at 250× *g* for 10 min to remove the debris. The supernatant was collected and incubated with the LDH reaction mixture at room temperature for 30 min in the dark. The released LDH levels were determined at 490 nm using a microplate reader (Molecular Devices).

### 2.5. In Vitro Scratch Migration Assay

CMT-U27 and CF41.mg cells were grown in 6-well plates. Confluent monolayer cells were scratched manually using a sterile 1000-μL pipette tip and gently rinsed with phosphate buffered saline. Subsequently, we added a fresh medium with different concentrations of esculetin to the wells. Following treatment, the images were photographed using a microscope at the indicated times (Nikon Eclipse TS100; Nikon Corporation, Tokyo, Japan).

### 2.6. Annexin-V/Propidium Iodide (PI) Staining

Cell apoptosis was assessed by flow cytometry using the annexin-V assay (Santa Cruz Biotechnology, Inc., Dallas, TX, USA) according to the manufacturer’s protocol. We determined the annexin-V content by measuring the fluorescence at 488 nm (excitation) and 525 nm (emission) using a Guava easyCyteHT system (Millipore, Billerica, MA, USA).

### 2.7. Terminal Deoxynucleotidyl Transferase dUTP Nick end-Labeling (TUNEL) Assay

Cells in the logarithmic phase were collected and cultured in 6-well plates at a density of 1 × 10^6^ cells/well. We assessed tumor cell apoptosis following treatment using an ApoBrdU DNA Fragmentation Assay Kit (BioVision, Milpitas, CA, USA) based on the manufacturer’s instructions. The nuclei were counterstained with propidium iodide (PI).

### 2.8. Western Blotting

The cells were homogenized in a cold lysis buffer containing protease inhibitor cocktail (Sigma-Aldrich). Each protein was separated by sodium dodecyl sulfate-polyacrylamide gel electrophoresis and transferred onto nitrocellulose membranes. The membranes were blocked with 5% skim milk and incubated with the following primary antibodies in a blocking buffer overnight at 4 °C: anti-cleaved caspase-3 (rabbit), cyclin-dependent kinase 4 (CDK4) (rabbit), anti-cyclin D1 (mouse) (all from Cell Signaling Technology, Inc., Beverly, MA, USA), and anti-β-actin (mouse; Sigma-Aldrich). Subsequently, the membranes were incubated with horseradish peroxidase (HRP)-conjugated secondary antibodies for 1 h at room temperature. Chemiluminescent signals were developed using an HRP substrate (Millipore) and detected using a Fusion FX7 acquisition system (Vilbert Lourmat, Eberhardzell, Germany).

### 2.9. Statistical Analysis

All data are represented as mean ± standard deviation (SD). Statistical significance between the groups were determined by the unpaired Student’s *t-*test. Significant differences in multi-group were assessed by a one-way analysis of variance, followed by Bonferroni post-tests. Statistical analysis was carried out by GraphPad Prism software. A *p*-value < 0.05 is considered to be significant.

## 3. Results

### 3.1. Esculetin Exerts Cytotoxic Effects on CMT Cells

**Figure 1:**
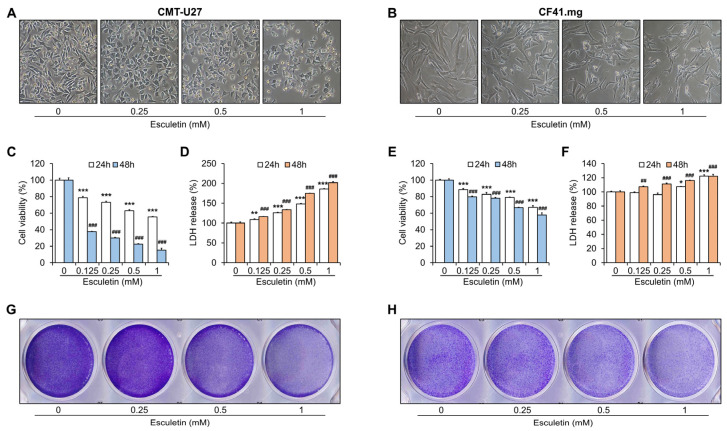
Esculetin inhibits the viability of CMT cells. (**A**,**B**) Images of morphological changes in CMT-U27 (**A**) and CF41.mg (**B**) cells after esculetin treatment. (**C**–**F**) Comparisons of the CMT-U27 (**C**,**D**) and CF41.mg (**E**,**F**) cell viability and LDH activity. (**G**,**H**) Images of crystal violet-stained cells in CMT-U27 (**G**) and CF41.mg (**H**) cells after esculetin treatment. Magnification, 100×. Values are mean ± SD. * *p* < 0.05, ** *p* < 0.01, *** *p* < 0.001 versus untreated cells at 24 h; ^##^ *p* < 0.01, ^###^ *p* < 0.001 versus untreated cells at 48 h by one-way ANOVA followed by Bonferroni post-test.

We first investigated the effects of esculetin on viability by MTT assay and LDH assay. Esculetin treatment in both CMT cells substantially decreased the number of viable cells (Figure 1A,B). The MTT assay revealed that esculetin inhibited the viability of both cell lines in a dose-dependent manner, compared with the controls (Figure 1C,E). The CMT-U27 cells displayed considerably reduced cell viability, which resulted in approximately 40% and 80% reduction at 0.5 mM following 24 h and 48 h of esculetin treatment, respectively. The cell viability gradually decreased in CF41.mg cells, thus reaching approximately 20% and 35% reduction at 0.5 mM following 24 h and 48 h, respectively. In the LDH assay, esculetin treatment significantly increased LDH release by 2-fold and 1.2-fold, compared with the control in the CMT-U27 and CF41.mg cells, respectively (Figure 1D,F). Furthermore, crystal violet-staining indicated that esculetin treatment decreased the cell viability in a dose-dependent manner (Figure 1G,H). Thus, esculetin exerted a potent cytotoxic effect against both CMT cell lines. On the other hand, to investigate the cytotoxic effects of esculetin on normal canine cells by themselves, we used canine aortic endothelial cells (CnAOECs) (Figure S1 (Supplementary Materials)). As a result, a slight inhibitory effect was observed when the CnAOECs were treated with esculetin for 24 h at lower than 0.5 mM, whereas 1 mM of esculetin considerably inhibited the CnAOECs’ viability.

### 3.2. Esculetin Suppresses Cell Migration in CMT Cells

**Figure 2:**
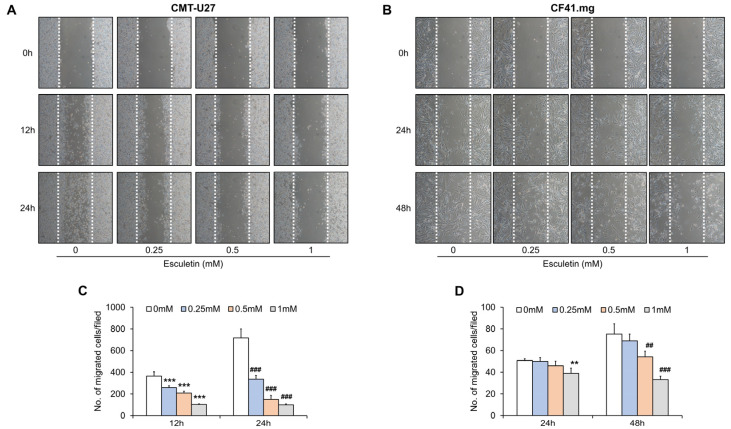
Esculetin suppresses migration of CMT cells. (**A**–**D**) Images and quantification of migration in CMT-U27 (**A**,**C**) and CF41.mg (**B**,**D**) cells after esculetin treatment in a dose- and time-dependent manner. Magnification, 40×. Values are mean ± SD. ** *p* < 0.01, *** *p* < 0.001; ^##^ *p* < 0.01, ^###^ *p* < 0.001 versus untreated cells by one-way ANOVA followed by Bonferroni post-test.

To determine the inhibitory effects of esculetin on cell migration, we performed a scratch migration assay. The number of migrated cells remarkably decreased with increasing concentrations of esculetin in CMT-U27 and CF41.mg cell lines (Figure 2). In CMT-U27 cells, cell migration decreased by 30%, 42%, and 70% (0.25 mM, 0.5 MM, and 1 mM, respectively) following 12 h, compared with treatment with 0 mM esculetin. Following 24 h, cell migration was considerably suppressed and reached 50%, 80%, and 90% (0.25 mM, 0.5 mM, and 1 mM, respectively) (Figure 2A,C). In CF41.mg cells, the number of migrated cells reduced with increasing doses of esculetin following 24 h; nonetheless, there was only a significant difference following treatment with 1 mM esculetin. However, we observed a considerable decrease in cell migration following 48 h. It reduced by 46% and 77% at 0.5 mM and 1 mM of esculetin, respectively (Figure 2B,D).

### 3.3. Esculetin Cause Apoptosis Via Caspase-3 Activation in CMT Cells

**Figure 3:**
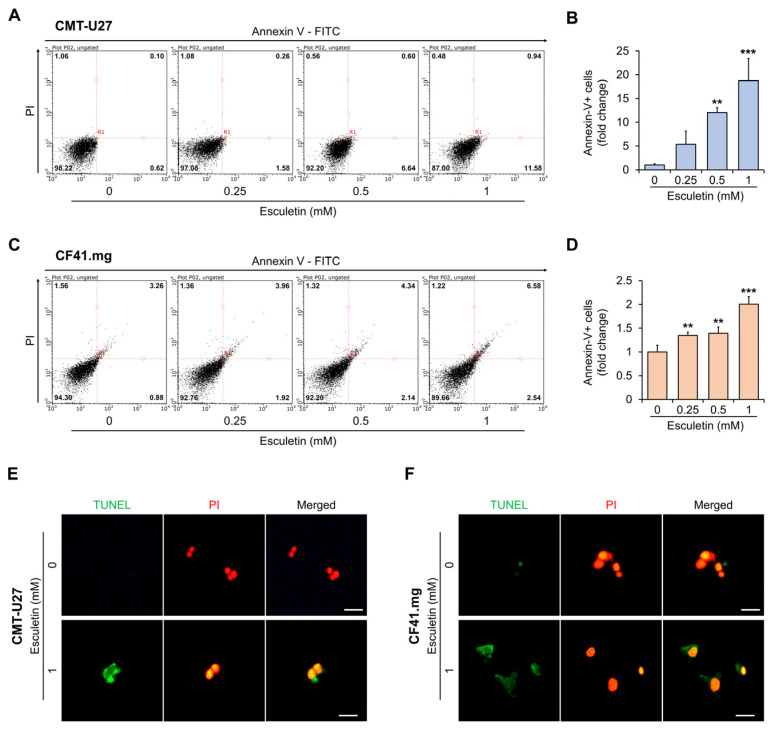
Esculetin induces apoptosis in CMT cells. (**A**–**D**) Flow cytometry analysis and quantification showing apoptosis in CMT-U27 (**A**,**B**) and CF41.mg (**C**,**D**) cells. (**E**,**F**) Fluorescent images showing an increase of TUNEL positive cells in CMT-U27 (**E**) and CF41.mg (**F**) cells. Scale bar, 25 μm. Values are mean ± SD. ** *p* < 0.01, *** *p* < 0.001 versus untreated cells by one-way ANOVA followed by Bonferroni post-test.

**Figure 4:**
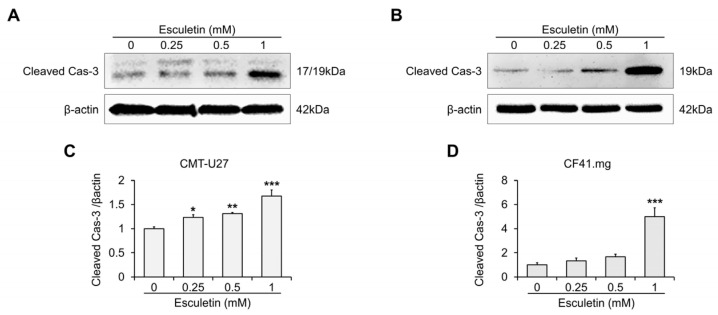
Esculetin induces apoptosis by activation of caspase 3 in CMT cells. (**A**–**D**) Images and quantification of the protein expression of cleaved cas-3 in CMT-U27 (**A**,**C**) and CF41.mg (**B**,**D**) cells after esculetin treatment. Values are mean ± SD. * *p* < 0.05, ** *p* < 0.01, *** *p* < 0.001 versus untreated cells by one-way ANOVA followed by Bonferroni post-test.

We subsequently explored if esculetin-induced cytotoxic effects occurred via apoptosis. Flow cytometric analysis revealed that the esculetin treatment increased the number of annexin-V-positive cells in a dose-dependent manner (Figure 3A–D). In particular, the proportion of apoptotic cells significantly increased by more than 10-fold and 1.3-fold in CMT-U27 and CF41.mg, respectively, at above 0.5 mM concentration, compared with the control. DNA fragmentation by esculetin was assessed using the TUNEL assay. We observed increased TUNEL expression in the cells treated with 1 mM esculetin following 24 h and 48 h (CMT-U27 and CF41.mg, respectively) (Figure 3E,F). Western blot analysis demonstrated that esculetin treatment gradually improved the protein expression of cleaved cas-3 in both CMT cell lines (Figure 4 and Figure S2). In particular, we confirmed a noticeable increase (approximately 5-fold at 1 mM concentration) in CF41.mg cells, whereas a 1.2- to 1.6-fold increase was observed in CMT-U27 cells treated with esculetin.

### 3.4. Esculetin Promotes Cell Cycle Arrest and Regulate Cell Cycle Related Proteins

**Figure 5:**
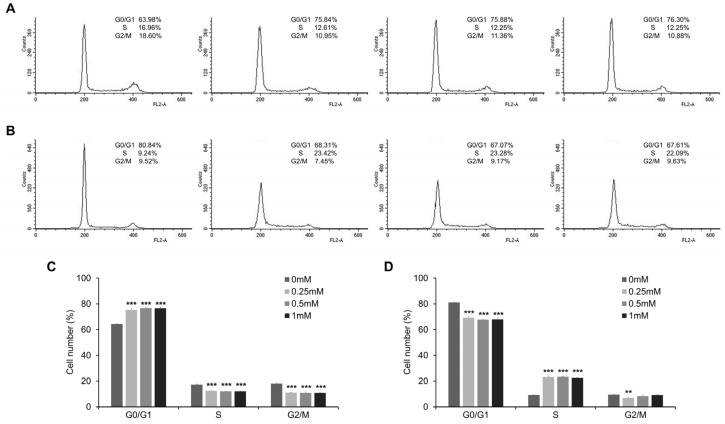
Esculetin induces cell cycle arrest at a different phase in CMT cells. (**A**–**D**) Cell cycle distribution and quantification in CMT-U27 (**A**,**C**) and CF41.mg (**B**,**D**) cells after esculetin treatment. Values are mean ± SD. ** *p* < 0.01, *** *p* < 0.001 versus untreated cells by one-way ANOVA followed by Bonferroni post-test.

**Figure 6:**
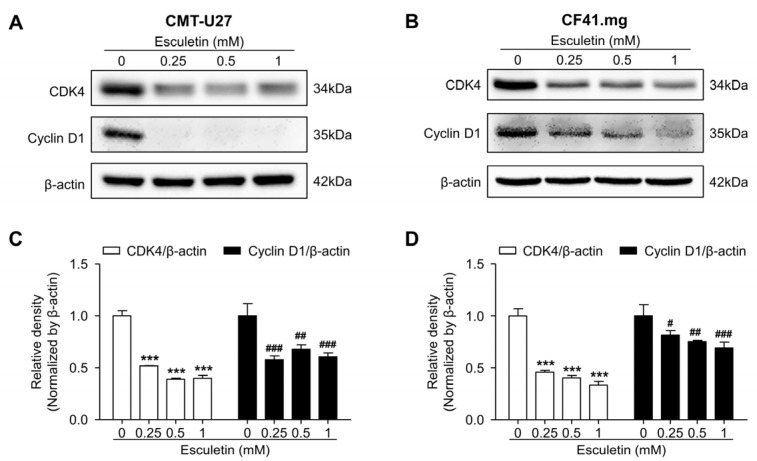
Esculetin downregulates the cell cycle-related proteins. (**A**–**D**) Images and quantification of the protein expression of CDK4 and cyclin D1 in CMT-U27 (**A**,**C**) and CF41.mg (**B**,**D**) cells after esculetin treatment. Values are mean ± SD. *** *p* < 0.001; ^#^ *p* < 0.05, ^##^ *p* < 0.01, ^###^ *p* < 0.001 versus untreated cells by one-way ANOVA followed by Bonferroni post-test.

We performed the flow cytometric analysis using PI to assess the effect of esculetin on cell cycle progression. Esculetin treatment induced cell cycle arrest at concentrations >0.25 mM. The G0/G1 phase distribution significantly increased in esculetin-treated CMT-U27 cells. Contrarily, the distribution of cells was reduced in the S phase, compared with that in the untreated groups (Figure 5A,C). In contrast, the G0/G1 phase distribution was significantly decreased in esculetin-treated CF41.mg cells. On the contrary, the S phase distribution was dramatically increased in esculetin-treated cells, compared with that in untreated cells (Figure 5B,D). In both CMT cell lines, the G2/M phase distribution was decreased more in esculetin-treated cells than in untreated cells. Western blot analysis revealed that esculetin promoted cell cycle arrest by downregulating cell cycle regulatory proteins, including CDK4 and cyclin D1 (Figure 6 and Figure S3). In CMT-U27 and CF41.mg cells, the relative intensities of CDK4 and cyclin D1 were remarkably lower in the esculetin-treated cells than those in untreated cells. Thus, esculetin-mediated cell cycle arrest might be one of the mechanisms underlying the antitumor effects of esculetin.

## 4. Discussion

Esculetin, a coumarin derivative, reportedly has several biological and pharmacological activities, including antioxidant, anti-inflammatory, and antiedema effects [22,29]. Moreover, recent studies have reported the anticancer effects of esculetin against various human cancers, including lung cancer [30,31], colorectal cancer [27,32,33], gastric cancer [34], and leukemia [35,36,37,38]. In particular, it is noteworthy in the antitumor effect of esculetin in human breast cancer, the counterpart of canine mammary cancer. Chang H.T. et al. reported that esculetin induced cytotoxicity by activating the Ca2+-associated mitochondrial apoptosis-signaling pathway in ZR-75-1 human breast cancer cells [39]. In addition, Jimenez-Orozco A.F. et al. proffered the significantly antiproliferative effects of esculetin by suppression of cyclin D1 expression in the MCF-7 human breast cancer cells [40]. However, no research on the effect of esculetin has been implemented in any type of canine cancer. In this novel study, we revealed the antitumor effects of esculetin on CMT-U27 and CF41.mg cell lines. Esculetin remarkably restrained the viability of both CMT cells, as determined by the MTT assay and crystal violet staining. Moreover, LDH release increased in esculetin-treated cells as compared to untreated cells, thereby indicating esculetin has potent cytotoxic activity against CMT cells.

Several studies evidenced the inhibitory effects of esculetin on cancer cell migration. Zhang et al. showed that esculetin decreased the migration of Hep-2 human laryngeal cancer cell lines by blocking the janus kinase/signal transducers and activators of transcription 3 signaling pathway [41]. Duan et al. demonstrated that esculetin remarkably attenuated the migration of 786-O and SN12-PM6 human renal-cell carcinoma cells. This ability of esculetin may be related to the reversal of the epithelial–mesenchymal transition progression, including the upregulation of E-cadherin expression [28]. Yan et al. revealed that esculetin suppressed the migration of HCT-116 colon cancer cells by inhibiting the wingless-related integration site/β-catenin signaling pathway. In addition, the combination of esculetin and 5-fluorouracil more potently decreased the HCT-116 cell migration, compared with 5-fluorouracil alone [32]. In this study, we performed a two-dimensional scratch assay to verify the inhibitory effects of esculetin on CMT cell migration. Esculetin dramatically attenuated cell migration in both CMT cell lines.

Numerous studies agree that induction of apoptosis and/or cell cycle arrest is the major approach in cancer therapy. In human cancer, several studies have proven that esculetin exerted cell cycle arrest. Duan et al. demonstrated that esculetin blocked cell cycle progression at the G0/G1 and G2/M phases in 786-O and SN12-PM6 renal cell carcinoma cell lines, respectively, which was caused by decreasing the expression levels of cyclin D1, CDK4, cell division protein kinase 6, and cellular myelocytomatosis oncogene [28]. Another study mentioned that esculetin not only upregulated the expression of p53, p27, and p21, it downregulated the expression of cyclin D1, which led to G0/G1 cell cycle arrest in LoVo human colon cancer cells [27]. In addition, esculetin suppressed the cell cycle at the G2/M phase in human oral cancer SAS cells [25] and in the S phase in human HCC SMMC-7721 cells [26]. The present study proved that esculetin suppressed the cell cycle progression in CMT cell lines. Interestingly, the protein expression of CDK4 and cyclin D1, regulators of G1/S transition, dramatically decreased in both CMT cells. However, esculetin caused cell cycle arrest at different phases in both CMT cell lines, i.e., at the G0/G1 phase in CMT-U27 cells and the S phase in CF41.mg cells. On the other hand, esculetin induced apoptotic cell death in both CMT cell lines, which was evidenced by the increased number of annexin-V-positive cells and induced DNA fragmentation. An increased cleaved caspase-3 protein also revealed that esculetin activated the caspase-dependent apoptosis. Collectively, these results showed that esculetin could induce apoptosis and cell cycle arrest by regulating related proteins in CMT cells.

Another interesting observation in this study is a discrepancy between the proportion of apoptotic cells and the protein expression of activated caspase-3. In Figure 3, at 1 mM esculetin, the proportion of apoptotic cells increased by approximately 18-fold in CMT-U27 cells and 2-fold in CF41.mg cells compared with the control. In contrast, in Figure 4, cleaved caspase-3 was increased approximately 5-fold at 1 mM esculetin-treated CF41.mg cells, whereas only a 1.6-fold in 1 mM esculetin-treated CMT-U27 cells, compared with the control. A possible explanation for this discrepancy might be differences between assays to determine apoptosis. Momoko Ariai et al. suggested that a discrepancy between bcl-2 expression and caspase-3 activity resulted from the difference in the apoptosis detection method [42]. Another study suggested that a discrepancy between caspase cleavage and cell death is due to the requirements in an assay for detecting caspase activity [43]. In our study, the results of annexin-V/PI staining showed that esculetin remarkably induced early apoptosis in CMT-U27 cells. However, caspases associated with early apoptosis were not addressed, only caspase-3, which is an executive caspase. In other words, it suggested that esculetin could affect other caspases such as caspase-9 in CMT-U27 cells. However, further studies are required to fully understand this discrepancy and the mechanisms of apoptosis in CMT cells.

## 5. Conclusions

In this study, esculetin significantly inhibited cell viability and migration through apoptosis and cell cycle arrest in CMT-U27 and CF41.mg cells. In conclusion, our findings evidence the antitumor effects of esculetin against CMT cells and lay a theoretical foundation for further studies including in vivo experiments and clinical trials for CMTs.

## Data Availability

Not applicable.

## Supplementary Materials

The following supporting information can be downloaded at: https://www.mdpi.com/article/10.3390/vetsci10020084/s1, Figure S1: Figure represents that the effect of esculetin on cell viability in canine aortic endothelial cells; Figure S2: Figure represents the uncropped scan and the densitometry readings/intensity ratio of each band of the Western blots shown in Figure 4A,B; Figure S3: Figure represents the uncropped scan and the densitometry readings/intensity ratio of each band of the Western blots shown in Figure 6A,B.
