# Supplementary material for: Antitumor Effects of Esculetin, a Natural Coumarin Derivative, against Canine Mammary Gland Tumor Cells by Inducing Cell Cycle Arrest and Apoptosis
Source: Vet Sci. 2023 Jan 22;10(2):84. doi: 10.3390/vetsci10020084 (PMC9961495; doi:10.3390/vetsci10020084)
Supplement: Supplementary file 1 [file vetsci-10-00084-s001.zip › vetsci-2096243-supplementary.pdf]

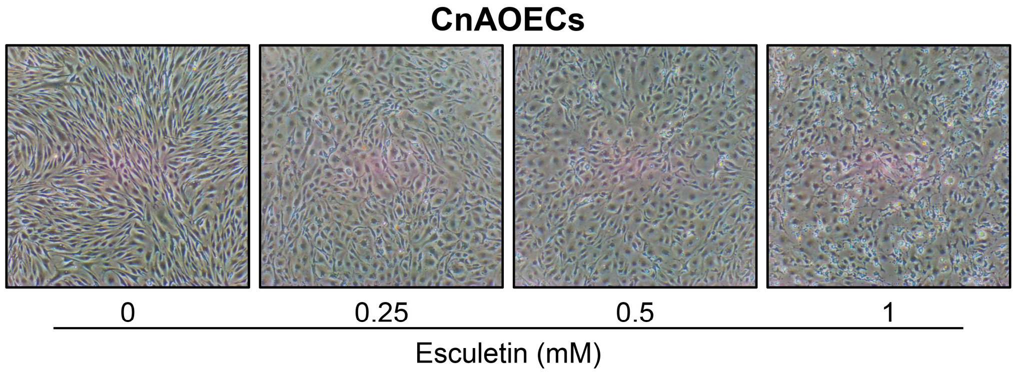

Figure S1. The effect of esculetin on cell viability in canine aortic endothelial cells. Canine aortic endothelial cells (CnAOECs) were treated with 0, 0.25, 0.5, and 1 mM of esculetin for 24 h. Following treatment, the cell images were photographed using a microscope (Nikon Eclipse TS100; Nikon Corporation, Tokyo, Japan).

a

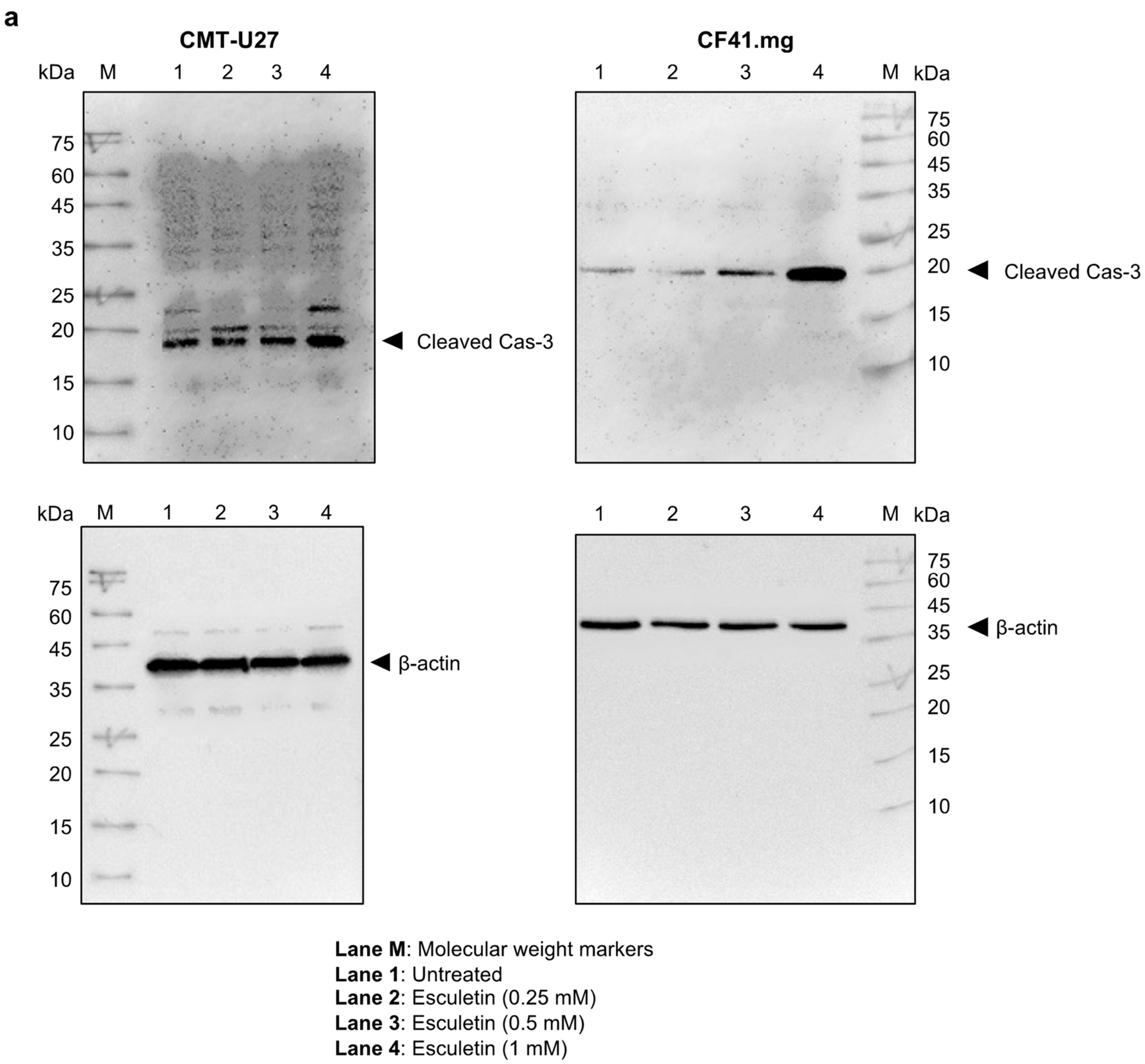

b

CMT-U27	
**Esculetin (mM)**	**Cleaved Cas-3/β-actin**
**0**	1.00 ± 0.04
**0.25**	1.23 ± 0.06
**0.5**	1.31 ± 0.02
**1**	1.68 ± 0.13

CF41.mg	
**Esculetin (mM)**	**Cleaved Cas-3/β-actin**
**0**	1.00 ± 0.18
**0.25**	1.34 ± 0.24
**0.5**	1.68 ± 0.21
**1**	5.00 ± 0.72

Figure S2. Immunoblots and densitometry reading/intensity ratio of apoptotic marker in CMT cells. CMT-U27 and CF41.mg cells were treated with 0, 0.25, 0.5, and 1 mM of esculetin for 24 h or 48 h. The protein expression of cleaved cas-3 (17/19 kDa) was analyzed by (a) western blots and (b) densitometry reading/intensity ratio. The band intensity was normalized to the corresponding β-actin value. Cas-3, caspase-3.

a

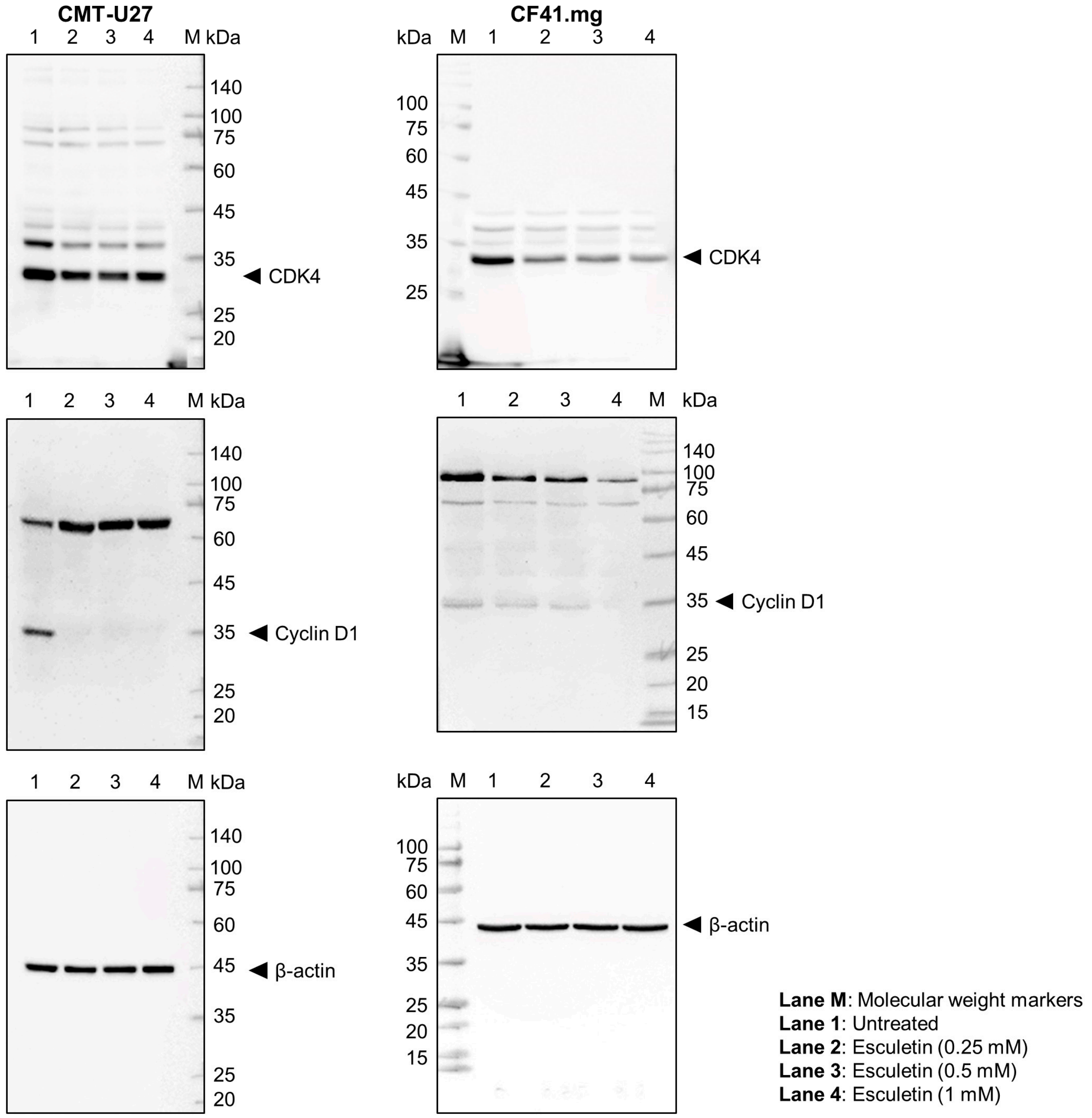

b

CMT-U27			CF41.mg		
**Esculetin (mM)**	**CDK4/β-actin**	**Cyclin D1/β-actin**	**Esculetin (mM)**	**CDK4/β-actin**	**Cyclin D1/β-actin**
**0**	1.00 ± 0.05	1.00 ± 0.12	**0**	1.00 ± 0.07	1.00 ± 0.11
**0.25**	0.52 ± 0.01	0.58 ± 0.03	**0.25**	0.46 ± 0.02	0.81 ± 0.04
**0.5**	0.39 ± 0.01	0.68 ± 0.04	**0.5**	0.40 ± 0.02	0.75 ± 0.01
**1**	0.40 ± 0.03	0.60 ± 0.04	**1**	0.33 ± 0.03	0.69 ± 0.06

Figure S3. Immunoblots and densitometry reading/intensity ratio of cell cycle related proteins in CMT cells. CMT-U27 and CF41.mg cells were treated with 0, 0.25, 0.5, and 1 mM of esculetin for 24 h or 48 h. The protein expression of CDK4 (34 kDa) and cyclin D1 (35 kDa) was analyzed by (a) western blots and (b) densitometry reading/intensity ratio. The band intensity was normalized to the corresponding β-actin value.
